# Perfluorooctane Sulfonate (PFOS) and Related Compounds Induce Nuclear Receptor 4A1 (NR4A1)-Dependent Carcinogenesis

**DOI:** 10.1021/acs.chemrestox.4c00528

**Published:** 2025-03-11

**Authors:** Amanuel Hailemariam, Srijana Upadhyay, Vinod Srivastava, Zahin Hafiz, Lei Zhang, Wai Ning Tiffany Tsui, Arafat Rahman Oany, Jaileen Rivera-Rodriguez, Robert S. Chapkin, Nicole Riddell, Robert McCrindle, Alan McAlees, Stephen Safe

**Affiliations:** † Department of Veterinary Physiology and Pharmacology, College of Veterinary Medicine, Texas A&M University, College Station, Texas 77843 , United States; ‡ Department of Veterinary Integrative Biosciences, Texas A&M University, College Station, Texas 77845 , United States; § Department of Nutrition, Program in Integrative Nutrition and Complex Diseases, Texas A&M University, College Station, Texas 77843 , United States; ∥ Wellington Laboratories Inc, 345 Southgate Dr., Guelph, ON N1G 3M5 , Canada

## Abstract

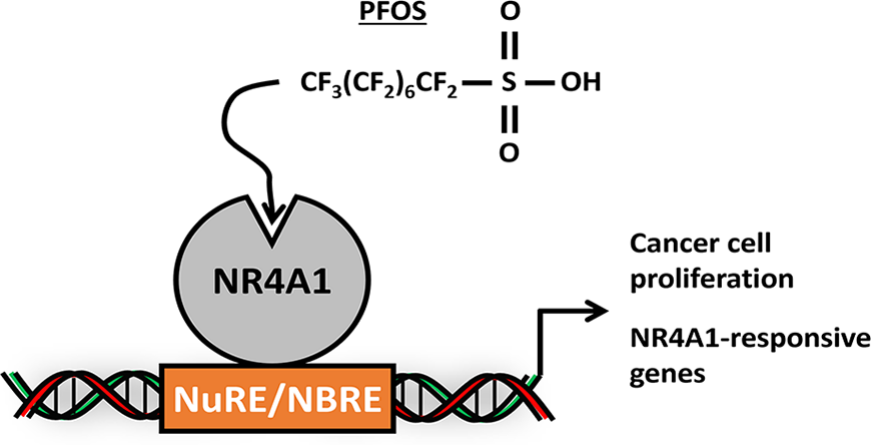

Polyfluoroalkyl substances (PFAS) are widely used industrial compounds that have been identified as contaminants in almost every component of the global ecosystem, and in human studies, higher levels of PFAS have been correlated with increased incidence of multiple diseases. Based on the results of human and laboratory animal studies, we hypothesize that the orphan nuclear receptor 4A1 (NR4A1) may be a critical target for some PFAS such as the legacy linear polyfluorooctanesulfonate (PFOS) and other sulfonates. We show that PFOS and related compounds bound the ligand binding domain (LBD) of NR4A1 and induced the growth of several cancer cell lines and enhanced tumor growth in an athymic nude mouse model. Using NR4A1-responsive rhabdomyosarcoma Rh30 cells as a model, PFOS induced NR4A1-dependent cell proliferation and Rh30 cell migration and invasion. Moreover, in Rh30 cells, PFOS also induces several NR4A1-regulated genes including the PAX3-FOXO1 oncogene and downstream gene products, and in a chromatin immunoprecipitation assay, PFOS does not decrease NR4A1 binding to the promoter. These results demonstrate that PFOS is an NR4A1 ligand and enhances tumorigenesis through the activation of this receptor.

## Introduction

Polyfluoroalkyl substances (PFAS) have been manufactured for over 80 years, and several thousand different individual PFAS have been synthesized for industrial, consumer, food packaging, and cosmetic applications.^1,2^ The widespread use of PFAS is due to several factors which include their thermal and chemical stability, water-repellent and flame-retardant activities, and amphipathic structures. The extensive production, use, and disposal practices of PFAS have resulted in their contamination of the global ecosystem including the marine and aquatic environments, fish, wildlife, food products, and humans.^3,4^ PFAS exposures are complex, and in humans, there is PFAS uptake from consumer products, food, environmental, cosmetic, and occupational exposures and contaminated water. Perfluorooctanoic acid (PFOA) and perfluorooctanesulfonate (PFOS) were among the most widely produced PFAS, and although uses of these compounds are restricted or have been eliminated, these legacy PFAS are still detected with high frequency in environmental and human samples.^3−8^ Several studies have investigated the association of PFAS levels in human serum samples with human diseases, and analysis of these studies showed that higher PFAS levels were associated with increased incidence of metabolic disease, endocrine disorders, cardiovascular disease, male and female reproductive tract problems, cancer, immune effects, urinary tract problems, and developmental toxicities.^8^ For example, individuals with high serum PFAS exhibit a higher incidence of multiple cancers including pancreatic, renal, thyroid, breast, and liver cancer.^9−23^ The mechanisms of PFAS-mediated responses are complex and dependent on the structure of the individual PFAS congener and the response. For example, in laboratory animal studies, administration of PFOS (5 or 10 mg/kg/day) to wild-type and humanized (PPARα) mice for 28 days resulted in changes in liver pathology and induction of ACOX1 and CYP4A11 enzymes that are consistent with a PPARα-dependent response, and induction of these genes was not observed in PPARα knockout mice. However, liver toxicities such as hepatomegaly that are induced by PFOS were PPARα -independent.^24^ There is also evidence that PFAS interacts with many other nuclear and cell surface receptors; however, direct linkages between these interactions and PFAS-associated adverse health effects are limited.^24−37^

Studies in this laboratory have identified a series of 1,1-bis(3′-indolyl)-1-(substitutedphenyl)methane analogues (CDIMs) that bind the pro-oncogenic orphan nuclear receptor 4A1 (NR4A1) and act as inverse NR4A1 agonists that inhibit NR4A1-dependent cancer cell growth and survival and enhance immune surveillance.^38,39^ For several NR4A1-dependent responses such as decreased immunity, enhanced neurotoxicity, metabolic disease, endometriosis, stress/inflammation, and cancer, the effects of CDIMs and other NR4A1 ligands^38−46^ on these responses are inversely related to those observed for epidemiologic studies which show the association between increased incidence of these disease with individuals exposed to higher levels of PFAS.^9−23^ For example, CDIMs act as inverse NR4A1 agonists and inhibit NR4A1-dependent cancer cell and tumor growth/viability,^38,47^ whereas some PFAS such as PFOS induce cancer cell and tumor growth/viability.^48,49^ Moreover, CDIMs downregulated the expression of the histone methyltransferase gene product G9a^45^ in cancer cell lines, whereas increased exposure to PFAS is associated with enhanced DNA methylation.^50−55^ Therefore, we hypothesize that NR4A1 plays a role in the toxicities associated with PFAS.

Previous laboratory studies show that some PFAS compounds enhance nontransformed breast and prostate cell growth and viability^48,49,53,56^ but do not induce cell transformation. In contrast, the effects of PFOS in vivo and in cell culture are dependent on the animal model, concentration of PFOS, and cancer cell context since inhibition or induction and no effects on cancer cell growth have been observed in studies using PFOS.^49,57−62^ This work investigates the effects of PFOS and structurally related compounds as NR4A1 ligands and NR4A1 agonists in cancer cells.

## Materials and Methods

### Cell Lines, Reagents, and Antibodies and Transactivation

Rh30 rhabdomyosarcoma, SW480, HCT116, RKO, and MC38 (mouse) colon cancer cells, MIA PaCa-2 pancreatic cancer cells, and CT26, U87MG, A172, and T98G glioblastoma cells were obtained from ATCC (Manassas, VA, USA). Cells were maintained in RPMI (St. Louis, MO, USA) medium supplemented with 10% FBS (Gibco/Invitrogen) at 37 °C in the presence of 5% CO_2_. Cells were treated with PFAS generously provided by Wellington Laboratories (Guelph, Ontario, Canada), and these include PFOS (technical grade), sodium perfluorooctanesulfonate (PFOS-XST), sodium perfluorohexanesulfonate (PFHxS-XST), sodium perfluoroheptanesulfonate (PFHpS-XST), sodium perfluorononanesulfonate (PFNS-XST), and sodium perfluorodecanesulfonate (PFDS-XST). The XST designation indicates that these compounds are linear and have been purified. Commercial PFOS contains some nonlinear impurities. The PPARγ inhibitor GW9662 was purchased from Tocris Biosciences (Minneapolis), and N-(4′-aminopyridyl-2-chloro-5-nitrobenzamide) (T007) was synthesized in the laboratory. The GAL4-NR4A1 chimera (LBD) and a UAS_5_-luc reporter construct were transfected into cancer cells, and induction of luciferase activity was determined as described.^43,46^

### Direct Binding Assay

At 25 °C, the Varian Cary Eclipse Fluorescence Spectrophotometer was used to examine the quenching of fluorescence of a Trp residue in the NR4A1 ligand binding domain to determine direct ligand binding.^65^ Different concentrations of PFAS ligands were incubated with the ligand-binding domain of NR4A1 (1.0 μM) in phosphate-buffered saline (PBS; pH 7.4). Wavelengths of excitation (at 285 nm with a slit width of 5 nm) and emission (between 300 and 420 nm with a slit width of 5 nm) were used to obtain fluorescence. Sigma Plot was used to perform data analyses. At a 330 nm emission wavelength, the concentration-dependent NR4A1 tryptophan fluorescence intensity was measured to quantify *R*^2^ and *K*_D_ values.

### Chromatin Immunoprecipitation (ChIP) Assay

The experimental protocol provided by the manufacturer was carried out using the ChIP-IT Express Kit (Active Motif, 53008). Rh30 cells were seeded on a plate for 24 h, then treated with DMSO, 10 μM PFOS, and 12.5 μM DIM-3,5-Cl_2_. After 24 h, treated cells were fixed and lysed, and nuclei were collected for shearing by sonication. Sheared chromatin samples were then immunoprecipitated overnight with antibodies using protein G-conjugated magnetic beads. NR4A1 antibodies and mouse IgG were used for the ChIP assay. Eluted chromatin was then purified using the Chromatin IP DNA Purification Kit (58002). Purified DNA was analyzed using amfiSure qGreen Q-PCR master mix (genDEPOT) for real-time PCR. The primers used for detection of the Human PAX3-FOXO1 promoter region were FOXO1 F 5′-TGCCTGTGCTTCACATTAGC-3′, FOXO1 R 5′-CAGATGGGGACAGAGACGC-3′, and G9a R 5′-CCCGGAGCATTGCACG-3′.

### Boyden Chamber (Micropore Membrane) Assay

Rh30 cells (2 × 10^5^) were seeded in RPMI medium supplemented with 2.5% charcoal-stripped fetal bovine serum prior to the 24 h treatment period. Subsequent treatment of cells was performed using different concentrations of PFOS for 24 h. Trypsinized counted cells (1 × 10^5^) were loaded in a BioCoat 8.0 μm 24-well plate with a growth factor reduced Matrigel invasion chamber from Corning (Bedford, MA). Cells were allowed to migrate for 48 h, followed by formaldehyde fixation and Crystal Violet staining. Migration of cells through the pores was quantified using ImageJ.

### Migration (Scratch-Wound) Assay

Rh30 cells (4 × 10^5^) were seeded and allowed to attach on 6-well plates for 24 h. RPMI medium was removed from the plates, and scratches were made using a sterile 200 μL pipet tip. PBS was used to wash and remove the dead cells. Attached cells were treated with either DMSO or different concentrations of PFOS (2.5 and 10 μM) in RPMI medium supplemented with 2.5% charcoal-stripped fetal bovine serum. The medium was removed and replaced with PBS after 24–48 h. Migrated cells were observed through the Evos digital inverted microscope, and images were taken to analyze the percent migration of Rh30 cells by using the ImageJ/Fiji wound healing size tool.

### Resazurin Proliferation Assay

Human Rh30 rhabdomyosarcoma cells were grown in RPMI medium. Cells were seeded in 96-well plates with a seeding density of 1.2 × 10^4^ cells per well. Cells were grown to ∼70% confluency and then treated with various concentrations of PFAS and other compounds as indicated. After 24 h, 0.02 mg/mL resazurin was added to each well and incubated for 4 h. End point fluorescent activity (excitation 540 nm and emission 590 nm) was measured as the reduction of resazurin to resorufin, an indicator for metabolic activity. The final concentration of DMSO in each well was 0.0032% to minimize DMSO-induced toxicity. Controls included on this plate included a vehicle control (DMSO) and an untreated control.

### Western Blotting

Rh30 cells (3 × 10^5^) were seeded and allowed to attach for 24 h on 6-well plates, followed by a 24 h treatment with either DMSO or different concentrations of PFOS. RIPA buffer that contained protease and phosphatase inhibitors was added to lyse cells, and 4–20% Mini-PROTEAN TGX Gels (BioRad, 4561094) were prepared to resolve whole-cell lysates. Polyvinylidene fluoride membrane was used to transfer proteins through wet blotting, blocked in 5% milk, followed by their incubation with primary and secondary antibodies. Protein bands, in the presence of Immobilon western Chemiluminescence HRP-substrates, were visualized using the BioRad ChemiDoc imaging system, and the antibodies were used as described: PAX3FOXO1 (C2944), G9a (C5688515), PARP (CS9532), N-Myc (SC2236) (Cell Signaling Technologies, Danvers, MA), c-Myc (SC-40) and β1-integrin (CS96995) (Santa Cruz, CA), TXNDC5 (GTX106914) (GeneTex, Irvine, CA) and NR4A1 (ab283264)(Abcam).^45,63,64^

### Small Interfering RNA Interference Assay

In six-well plates, Rh30 cells (1.5 × 10^5^) were seeded and allowed to reach approximately 60% confluency in 24 h. Lipofectamine RNAiMAX was used for cell transfection. A transfection mixture prepared with siRNA along with Lipofectamine RNAiMAX Reagent (Invitrogen; 56531) and Opti-MEM (Gibco; 31985–062) following the Lipofectamine RNAiMAX reagent protocol was used after 24 h. Replacement of the Opti-MEM with fresh medium was performed after 6 h of transfection, and cells were incubated (at 37 °C, 5% CO_2_) for an additional 72 h. Harvested cells were used to determine the expression of proteins and RNA analysis. Western blots were performed to determine the efficiency of NR4A1 knockdown by siRNAs targeting NR4A1 that were purchased from Sigma-Aldrich. siRNAs used were siNR4A1 (NR4A1_C and NR4A1_D and Scrambled siRNA (CGU ACG CGG AAU ACU UCG A (Sigma-Aldrich).

### Animal Studies

The animal study protocols were approved by the Institutional Animal Care and Use Committee (IACUC) at Texas A&M University. Four-week-old male athymic nude mice were obtained from The Jackson Laboratory (Bar Harbor, ME) and housed at the Laboratory Animal Resources and Research facility, Texas A&M University. Male mice were chosen for this study based on their enhanced responsiveness to PFOS in a preliminary study; future research will confirm male vs female responsiveness to PFAS using xenograft and syngeneic mouse models. Mice were allowed to acclimate for 1 week and were fed a standard chow diet. Each mouse received an injection of 2 × 10^6^ Rh30 cells suspended in 100 μL of a 1:1 Matrigel and PBS solution into each flank subcutaneously. Once tumors reached a palpable size (approximately 50 to 100 mm^3^), the mice were randomly assigned to control and treatment groups. Mice in the control group were administered 100 μL of a DMSO:corn oil (1:4) solution by oral gavage daily. Mice in the treatment groups were administered 100 μL of a PFOS solution prepared in DMSO:corn oil (1:4) by oral gavage daily at doses of 20, 10, 0.5, and 0.2 mg/kg/day. The mice were weighed regularly, and where possible, their tumor volumes were measured using a Vernier Caliper (*V* = *L* × *W* × *H* mm^3^) every week. After 4 weeks of drug administration, the mice were euthanized, and their tumors were excised and weighed. A portion of each tumor was homogenized in lysis buffer, and the resulting extract was used for Western blot analysis.

### Statistical Analysis

Statistical analysis was conducted using the *t* test to assess differences between the groups. To compare the median survival rates of tumor-bearing animal cohorts, the log-rank (Mantel-Cox) test was applied with the analysis performed using Prism 9 software. All in vitro experiments were repeated three times to ensure the reliability and consistency of results. In vitro results are presented as the ± SD, and in vivo results are means ± SE. To determine statistical significance, a one-way analysis of variance (ANOVA) with Dunnett’s posthoc test was used, and a *P* value <0.05 was considered statistically significant.

## Results

**Figure 1:**
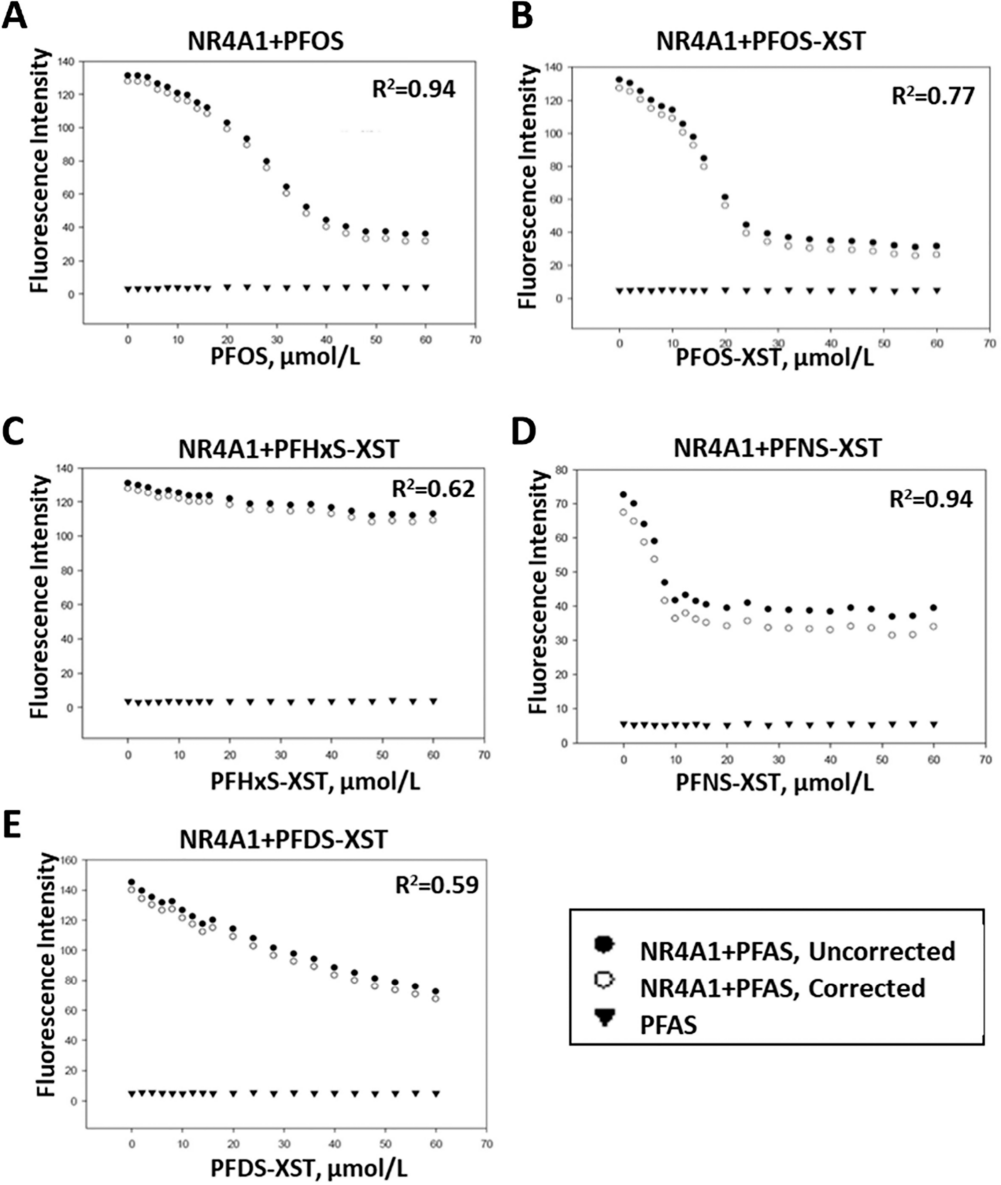
PFAS compounds bind to NR4A1. PFOS (A), PFOS-XST (B), PFHxS-XST (C), PFNS (D), and PFDS (E) were incubated with the ligand binding domain of NR4A1, and the binding curves were generated as outlined in the Materials and Methods section. Results obtained for the compounds alone (▼), ligand plus receptor uncorrected (●), and (ligand + receptor)–(ligand alone) corrected (○). The *K*_D_ values were determined for all compounds that bound NR4A1 and not PFHxS-XST which exhibited minimal quenching of fluorescence; the *K*_D_ values were 2.99 (PFOS), 0.92 (PFOS-XST), 0.24 (PFNS-XST), and 9.12 (PFDS-XST) μmol/L.

PFOS and some analogues are legacy PFAS compounds detected in most human samples and were chosen as models for this study. Initial studies examined the binding of PFOS and structurally related compounds to the ligand binding domain (LBD) of NR4A1 using a fluorescent assay which measures quenching of the fluorescence of a Trp residue in the LBD of NR4A1 as previously described.^65^Figure 1 summarizes the binding curves generated from PFAS ligands and their interactions with the LBD of NR4A1. Commercial PFOS (Figure 1A), the linear PFOS-XST (Figure 1B), the linear hexafluoro (PFHxS-XST; Figure 1C), linear nonafluoro (PFNS-XST; Figure 1D), and decafluoro (PFDS-XST; Figure 1E) alkyl sulfonates differentially decreased fluorescence associated with a Trp residue in the LBD. Only minimal displacement was observed for PFHxS which was considered to be inactive in the quenching assay. The KD values observed for binding of PFOS, PFOS-XST, PFNS-XST, and PFDA-XST were 2.99, 0.92, 0.24, and 9.12 μmol/L, respectively. The commercially available PFOS contains some branched PFOS isomers; however, the binding and *K*_D_ values were similar to those of the purified linear PFOS-XST.

**Figure 2:**
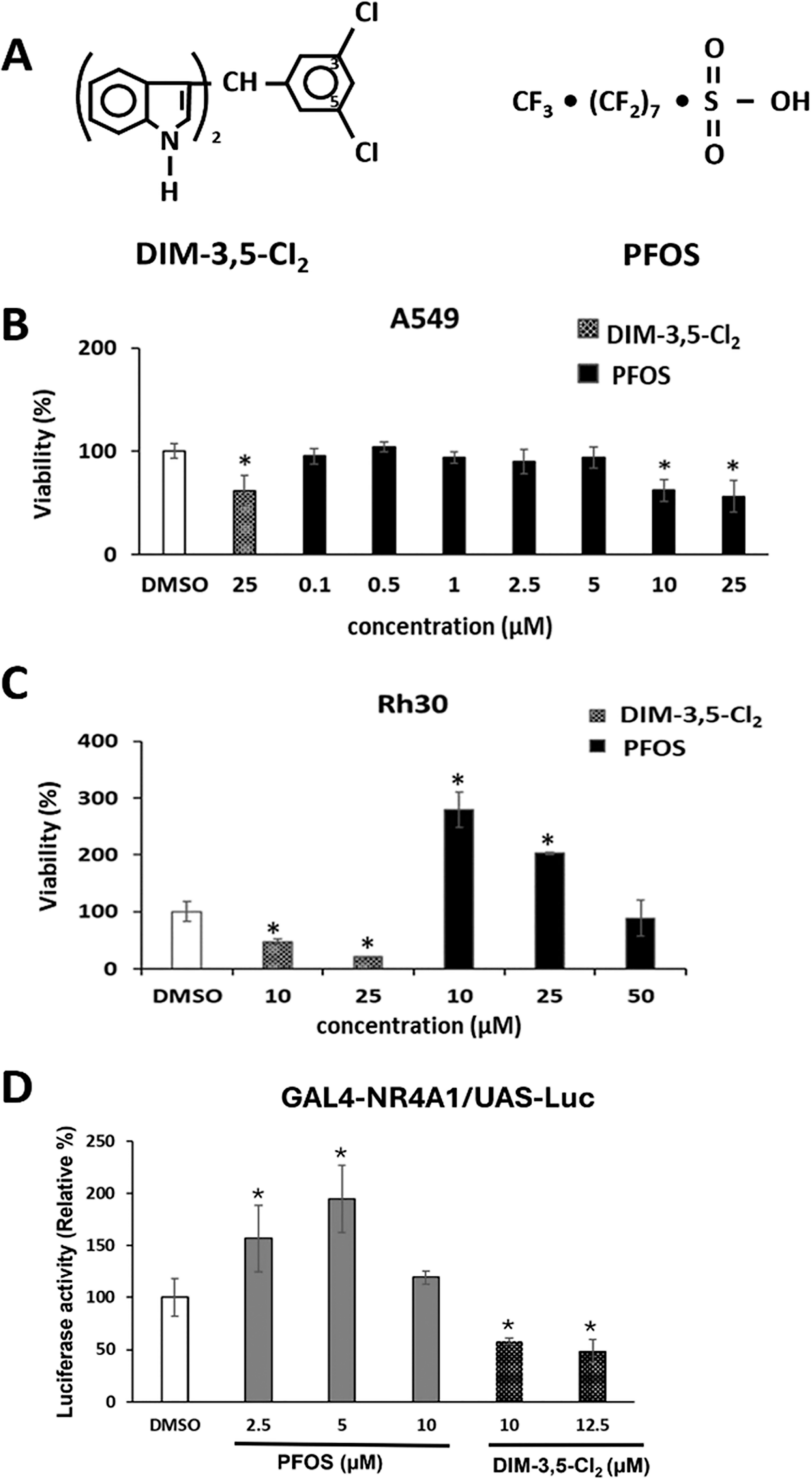
Comparative induction and growth-promoting effects of DIM-3,5-Cl_2_ and PFOS. (A) Structures of DIM-3,5-Cl_2_ and PFOS. Comparative effects of DIM-3,5-Cl_2_ and PFOS on the growth of A549 lung cancer (B) and Rh30 rhabdomyosarcoma (C) cells after treatment for 24 h. (D) Rh30 cells were transfected with GAL4-NR4A1 and UAS-Luc plasmids, and after treatment with PFOS or DIM-3,5-Cl_2_ luciferase activity was determined as outlined in the Methods. Results are expressed as means ± SD for at least 3 replicates for each treatment groups, and significant (*p* < 0.05) induction or inhibition of growth or luciferase activity is indicated (*).

Previous studies have associated human exposures to higher levels of PFOS with increased levels of cancer, whereas in cancer cell lines, the growth-promoting activities of PFAS are highly variable. For example, PFOS alone at low doses (10^–10^ – 10^–5^ M) did not affect T47D breast cancer cell growth, but higher concentrations (>10 ^–5^ M) inhibited growth.^58^ In A549 lung cancer cells, there was a >10% increase in cell proliferation by 50 and 100 μM PFOS, and cytotoxicity was observed at higher concentrations (200–1000 μM).^66^ In this study, we compared the cytotoxicity of the inverse agonist 1,1-bis(3′-indolyl)-1-(3,5-dichlorophenyl)methane (DIM-3,5-Cl_2_) and PFOS in A549 cells (Figure 2A). DIM-3,5-Cl_2_ (25 μM) inhibited A549 cell viability, and this has previously been observed for CDIM compounds in lung and other cancer cell lines where the CDIMs inhibit NR4A1-dependent growth^67^ (Figure 2B). In contrast, 100 nM–5 μM PFOS did not increase cell viability, whereas higher concentrations (10 and 25 μM) inhibited the growth of A549 cells as previously reported.^57^ This experiment was repeated in Rh30 rhabdomyosarcoma cells and DIM-3,5-Cl_2_ (10 and 25 μM) inhibited cell growth as previously reported for CDIMs,^47,63,64^ whereas 10 and 25 μM PFOS induced >2.5-fold increase in Rh30 cell proliferation over a 24 h treatment period (Figure 2C). In addition, PFOS induced and DIM-3,5-Cl_2_ decreased the luciferase activity in Rh30 cells transfected with GAL4-NR4A1 and UAS-Luc constructs. These observations demonstrate that PFOS induces Rh30 cell proliferation and NR4A1-dependent transactivation. Induction of cell growth by PFOS was cell context-dependent in A549 and Rh30 cells. In contrast, DIM-3,5-Cl_2_ decreased the proliferation of both A549 and Rh30 cells, and the growth-promoting effects of PFOS were inversely related to the growth-inhibiting effects of DIM-3,5-Cl_2_.

**Figure 3:**
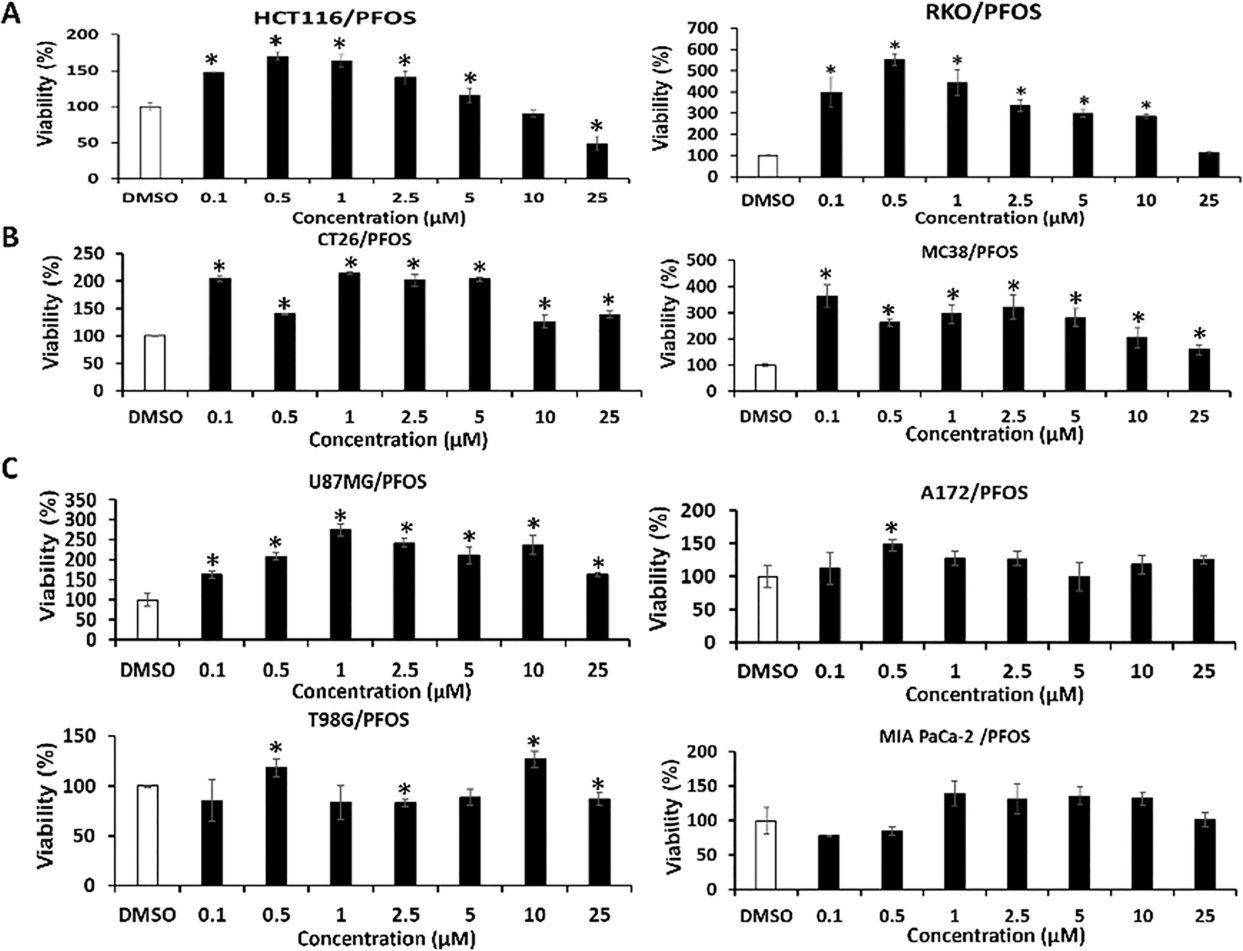
PFOS inducing cancer cell proliferation screening. HCT116 and RKO (A), CT26 and MC38 (B), U87MG, and A172 (C), and T98 and MIA PaCa-2 (D) were treated with PFOS (0.1–25 μmol/L) for 24 h, and cell proliferation was determined using the resazurin assay as outlined in the Methods. Results are expressed as means ± SD for at least 3 replicates for each treatment groups, and significantly (*p* < 0.05) increased or decreased growth is indicated (*).

We further examined the effects of several polyfluorinated alkyl compounds on the growth of 8 different cancer cell lines using a range of concentrations from 0.1 to 25 μM (Figure 3). The results show that for a number of cancer cell lines, PFOS induced a >2-fold increase in RKO, CT26, MC38, and U87 cell growth, whereas a <2-fold increase was observed in HCT116, A172, T98G, and MIA PaCa-2 cells. PFOS induced some proliferation of most cancer cell lines; however, the responsiveness of these cells was variable. One possible explanation for the different responsiveness of cancer cell lines to PFOS-induced cell proliferation may be due to the expression of PPARγ which also binds PFOS. Since PPARγ ligands primarily inhibit cancer cell growth, we cotreated Rh30 and A549 cells treated with PFOS alone and in combination with the PPARγ inhibitors T007 and GW9662 expecting that by blocking PPARγ, PFOS-induced growth would be enhanced. The results showed the PPARγ inhibitors had minimal effects on PFOS-induced growth of Rh30 and A549 cells, and therefore, PPARγ expression was not related to the cell context-dependent growth-promoting effects of PFOS. Interestingly, the >3-fold induction of growth by PFOS in HCT116, RKO, MC38, and U87G cells is unusually high and exceeds the effects of most growth factors in cancer cells.

**Figure 4:**
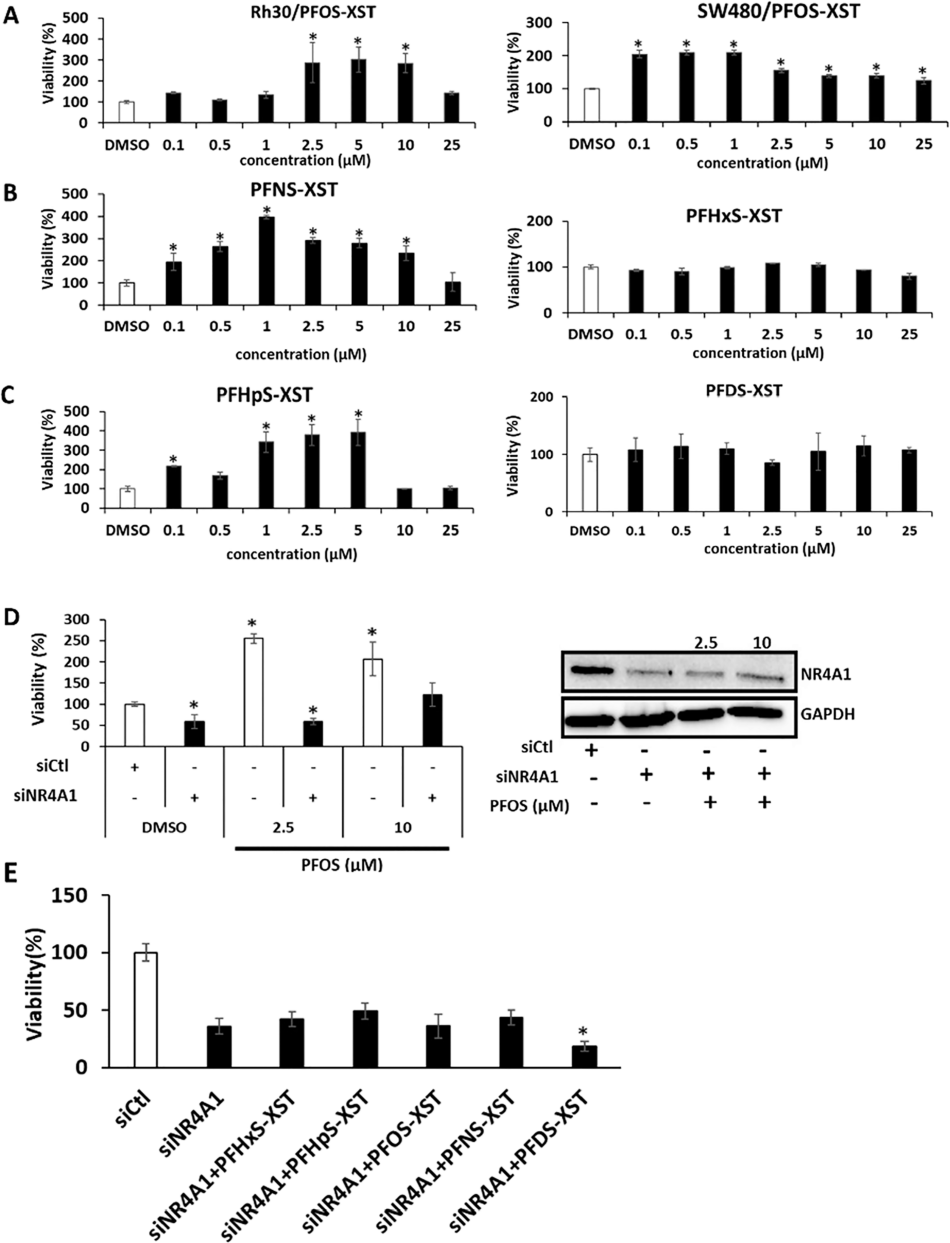
Induction of Rh30 cell growth by polyfluoroalkyl sulfonates. Rh30 and SW480 (A) cells were treated with PFOS-XST for 24 h, and cell viability was determined as outlined in the Materials and Methods section. Rh30 cells were treated with PFNS-XST and PFHxS-XST (B) and PFHpS-XST and PFDS-XST (C) for 24 h, and cell viability was determined as outlined in the Materials and Methods section. (D) Rh30 cells were transfected with siCt1 or siNR4A1 and treated with 2.5 or 10 μM PFOS, and cell proliferation and Western blot analyses on whole cell lysates were determined as outlined in the Methods. (E) Cells were also transfected with siNR4A1 alone and after treatment with 2.5 μM polyfluoroalkyl sulfonates, and cell viability was determined as outlined in the Methods. Results are expressed as means ± SD for at least 3 replicate determinations for each treatment group, and significant (*p* < 0.05) induction or inhibition is indicated (*). The XST designation for these compounds indicates that they are purified linear polyfluoroalkyl sulfonates.

The effects of PFOS and structurally related sulfonates on the growth of cancer cells were investigated over a broad range of concentrations (Figure 4A). PFOS significantly induced the proliferation of Rh30 cells at concentrations between 2.5 and 10 μM, whereas in SW480 cells, PFOS concentrations as low as 100 nM and as high as 25 μM significantly induced SW480 cell proliferation, indicating that the SW480 cell line was also highly responsive to the growth-promoting activity of PFOS. We also examined the effects of a series of perfluoroalkyl sulfonates containing 9 and 6 (Figure 4B) and 7 and 10 (Figure 4C) carbon atoms on the proliferation of Rh30 cells. This cell line was chosen as a model since previous studies show that it is NR4A1-responsive with respect to cell growth and related pro-oncogenic pathways/genes.^45−47,63^ Both the nona- and hepta-compounds (PFNS and PFHpS) enhanced cell proliferation, whereas this was not observed for the deca- and hexa- (PFDS and PFHxS) sulfonates. The lack of activity for PFDS was surprising based on the binding data for this compound which exhibited a low *K*_D_ value and significant fluorescence quenching in the receptor binding assay, whereas PFHxS had minimal effects on cell growth and exhibited minimal binding in the fluorescence quenching assay (Figure 1). The maximal magnitude of growth enhancement by the active polyfluoroalkyl sulfonates varied from 2- to 4-fold in Rh30 cells, and the magnitude of this response was greater than the effects previously observed in Rh30 cells for transforming growth factor β in previous studies.^47,63,64^ Results in Figure 4D show that knockdown of NR4A1 (siNR4A1) decreased the growth of Rh30 cells, and in the NR4A1-deficient cells, induction of growth by 2.5 or 10 μM PFOS was inhibited. The efficiency of NR4A1 knockdown is shown in Western blot (Figure 4D). This confirms a role for NR4A1 in mediating the growth-promoting effects of PFOS, and results in Figure 4E show that knockdown of NR4A1 also blocks the growth-promoting effects of several structurally related perfluoroalkyl sulfonates. These results suggest that the perfluoroalkyl sulfonates act as NR4A1 agonists to enhance NR4A1-dependent proliferation responses.

**Figure 5:**
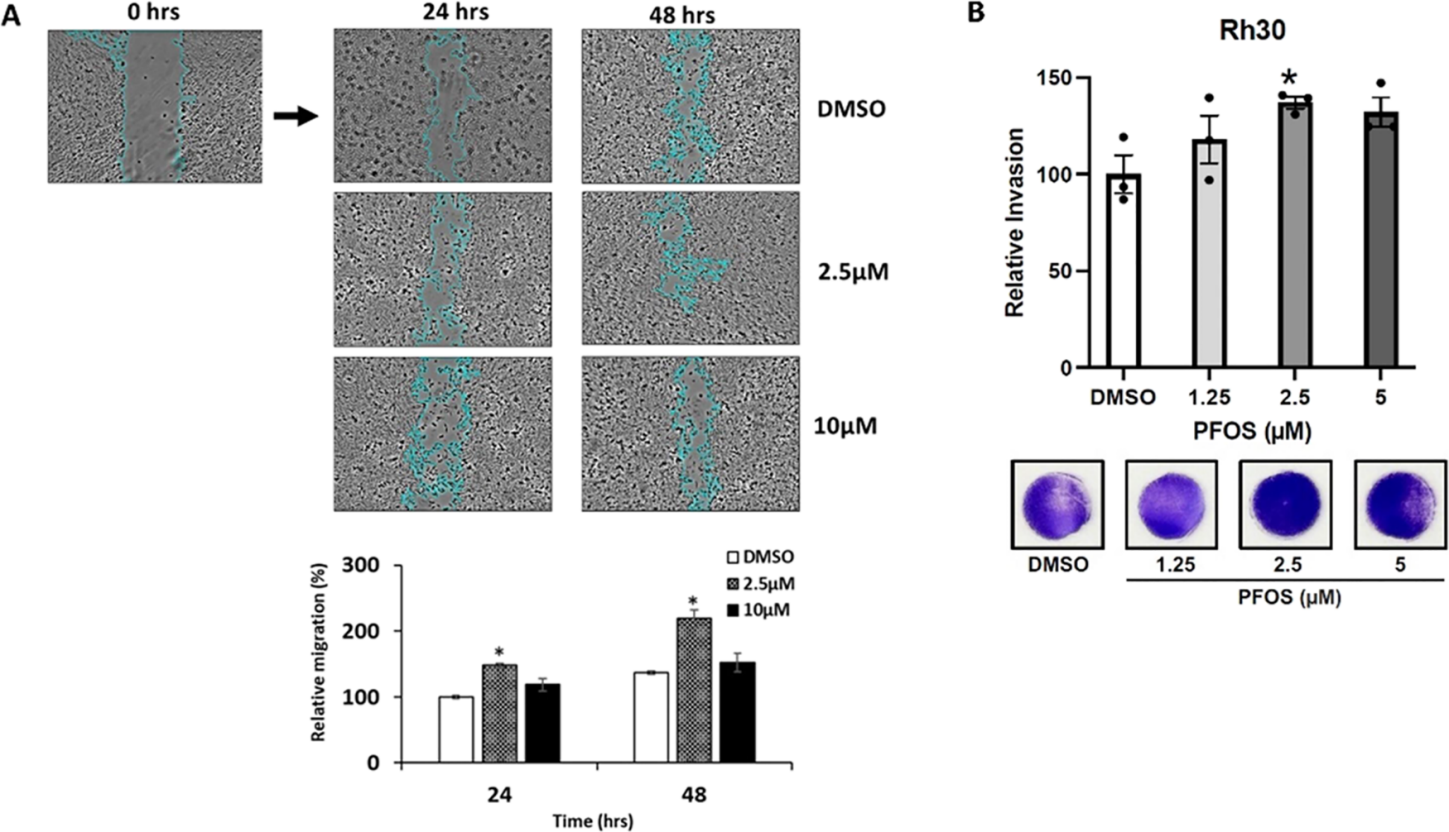
PFOS induces cell migration. (A) Rh30 cells were treated with DMSO (control) 2.5 or 10 μM PFOS for 24 h, and cell migration was determined in scratch assay and quantitated as outlined in the Materials and Methods section. A Boyden chamber assay (B) was also carried out in Rh30 cells as outlined in the Methods. The assays were carried out in triplicate; results are expressed as means ± SE, and significantly (*p* < 0.05) enhanced migration is indicated (*).

**Figure 6:**
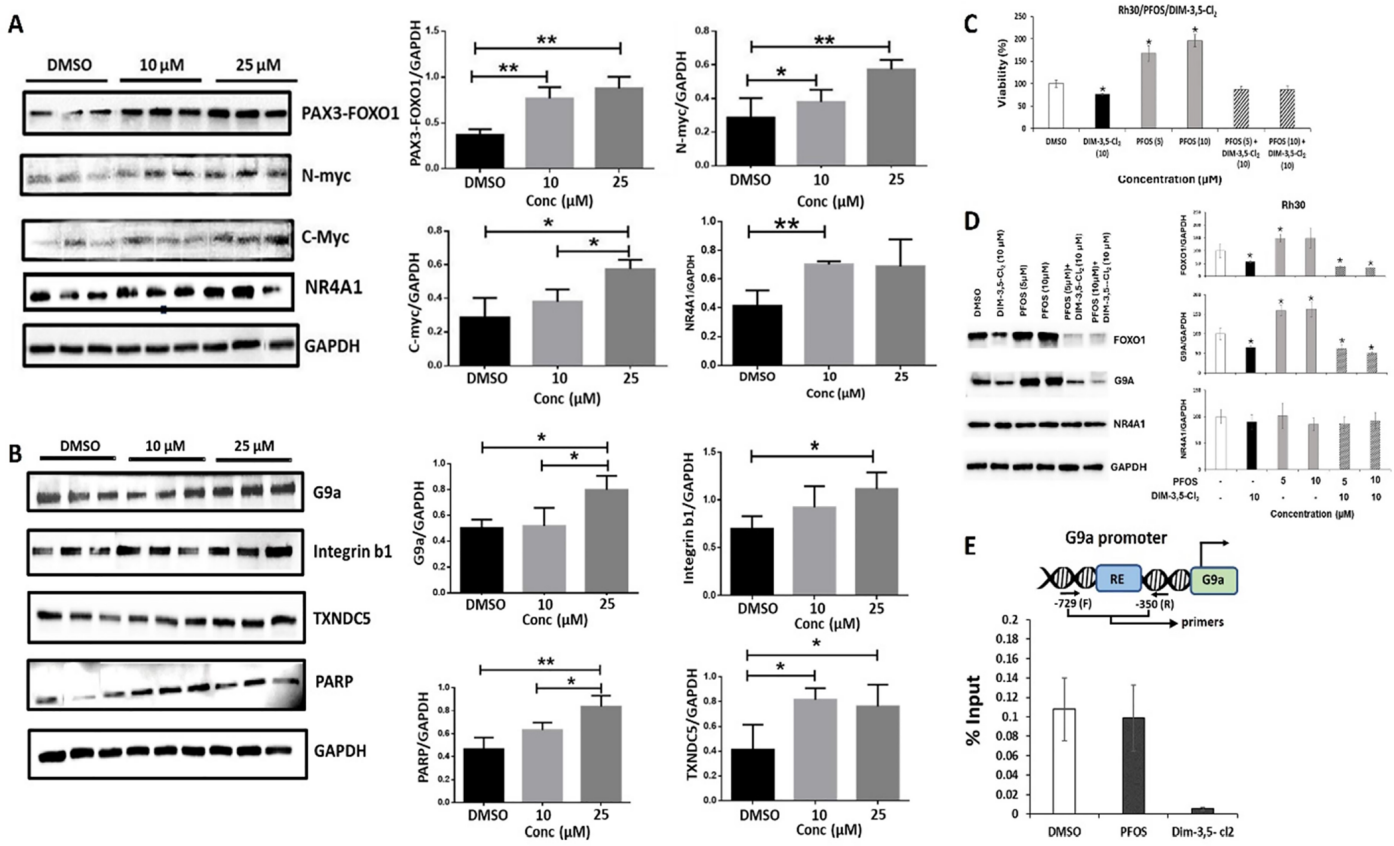
PFOS induces NR4A1-dependent gene products in the Rh30 cells. Rh30 cells were treated with 10 or 2.5 μM PFOS for 24 h, and whole cell lysates were analyzed for PAX3-FOXO1, N-Myc, and c-Myc (A) and other NR4A1-regulated gene products (B) by Western blots. Rh30 cells were treated with PFOS and DIM-3,5-Cl_2_ alone or in combination and effects on cell proliferation (C), and gene products (D) were determined as outlined in the Methods. (E) Cells were treated with DMSO (control) PFOS (10 μM) and DIM-3,5-Cl_2_ (12.5 μM), and interactions of NR4A1 with the G9a gene promoter were determined in a ChIP assay as outlined in the Methods. The Western blots were carried out in triplicate, and band intensities (means ± SD) were determined relative to GAPDH (control), and significant (*p* < 0.05) induction or inhibition is indicated (*).

The effects of PFOS on several NR4A1-regulated responses in Rh30 cells were investigated, and this includes their effects on Rh30 cell migration using a scratch assay in which cells were treated with DMSO (control) and 2.5 or 10 μM PFOS for 24 and 48 h. The results showed that the relative migration of Rh30 cells was increased after treatment for 24 or 48 h (Figure 5A); however, significant induction of cell migration was only observed for the 2.5 μM dose. Results in Figure 5B show that 2.5 μM PFOS enhances the invasion of Rh30 cells in a Boyden chamber assay, and this complements the enhanced migration observed in the scratch assay. In previous studies, the CDIM/NR4A1 inverse agonists modulated the expression of several NR4A1-regulated gene products in Rh30 cells, and these include the PAX3-FOXO1 fusion oncogene, c-Myc and N-Myc.^47,63,64^ Results illustrated in Figure 6A show that 10 or 25 μM PFOS induces levels of PAX3-FOXO1 and N-Myc proteins in Rh30 cells and 25 μM PFOS also induces c-Myc levels in this cell line. In addition, treatment of Rh30 cells with 10 or 25 μM or both concentrations of PFOS for 24 h also increased levels of several other NR4A1-regulated gene products including G9a (25 μM), β1-integrin (25 μM), thioredoxin domain containing 5 (TXNDC5) (10 and 25 μM), and PARP cleavage (10 and 25 μM). DIM-3,5-Cl_2_ acts as an inverse NR4A1 agonist in cancer cells, and in Rh30 cells, induction of proliferation by PFOS is inhibited by DIM-3,5-Cl_2_ (Figure 6C). Moreover, DIM-3,5-Cl_2_ also inhibits PFOS-induced NR4A1-regulated PAX3-FOXO1 (FOXO1) and G9a gene products in Rh30 cells (Figure 6D). Previous studies in this laboratory showed that CDIM compounds decreased interactions of NR4A1 with the transcriptionally active region of the G9a gene promoter in a ChIP assay.^45^ Results in Figure 6E also show that after treatment with 10 μM PFOS, there is no change in NR4A1 interactions with the G9a promoter compared to that observed in the untreated cells, whereas DIM-3,5-Cl_2_ (12.5 μM) significantly decreased NR4A1-G9a gene promoter interactions. This is another example of the inverse relationship between the effects of PFOS and CDIMs, which is also observed for Rh30 cell proliferation, migration/invasion, and gene product expression, indicating that PFOS is acting as an NR4A1 agonist.

**Figure 7:**
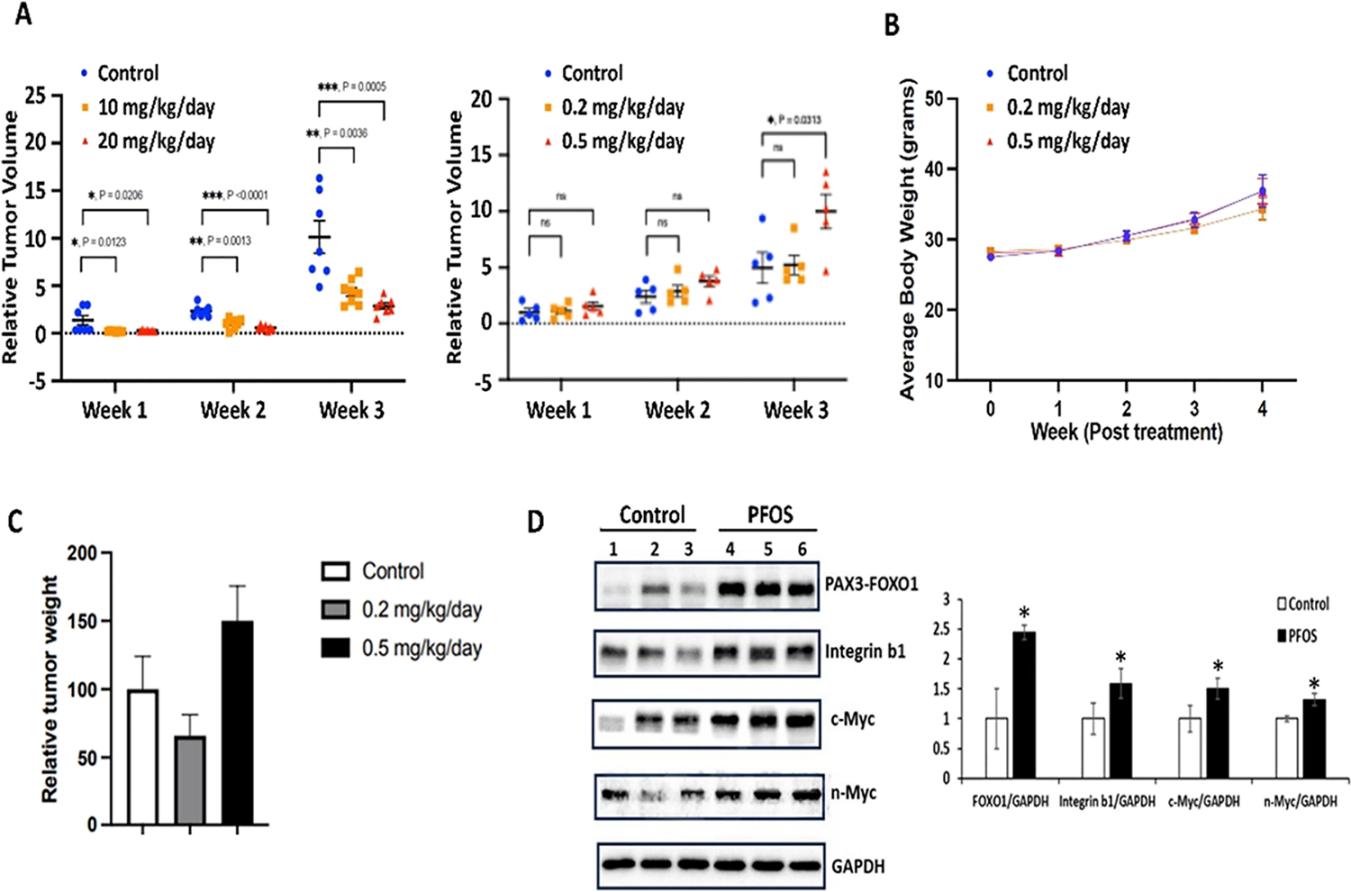
In vivo studies. Athymic nude mice bearing Rh30 cells were treated with 20, 10, 0.5, and 0.2 mg/kg/day and tumor volumes (A), body weight (B), relative tumor weights (C), and Western blot analysis of tumor lysates (D) were determined as outlined in the Materials and Methods section. Results (A–D) are expressed as means ± SE, and significant (*p* < 0.05) induction is indicated (*).

In initial studies, it was observed that higher concentrations of PFOS (10 and 20 mg/kg/day) significantly inhibited tumor growth in an athymic nude mouse model using Rh30 cells as xenografts (Figure 7A). The doses of PFOS were then lowered to 0.2 and 0.5 mg/kg/day and tumor volumes were observed over a period of 4 weeks after injection of the cells. A summary of the results demonstrates that after 3 or 4 weeks of treatment with 0.5 but not 0.2 mg/kg/day PFOS, there was a significant induction of tumor volumes compared to the control (corn oil-treated) mice (Figure 7A). Body weights were not significantly different between the control and PFOS-treated mice (Figure 7B), and while relative tumor weights were increased in the 0.5 mg/kg/day treatment group, the effect was not significantly different than the controls or mice treated with 0.2 mg/kg/day PFOS (Figure 7C) due to interindividual animal variability. Results in Figure 7D show that in tumor lysates from the 0.5 mg/kg/day treatment group levels of NR4A1-responsive genes were significantly induced compared to controls, and this further supports that PFOS is acting through NR4A1.

## Discussion

PFOS is an important legacy PFAS compound that is routinely identified as a major PFAS component in environmental samples and human serum. Although PFOS induces multiple responses in cell culture and laboratory animals, the collective effects of PFOS and related PFAS in humans indicate that higher levels of these compounds are associated with an increased incidence of several diseases. As indicated in the Introduction, diseases associated with exposures to high levels of PFAS have a linkage to NR4A1 and these adverse responses are ameliorated after treatment with an NR4A1 ligand such as celastrol, cytosporone B and related compounds, and CDIMs.^38−46^ For example, CDIM ligands act as inverse receptor agonists that inhibit NR4A1-dependent pro-endometriotic and pro-carcinogenic responses,^42,43^ whereas higher PFAS levels in humans are associated with increased endometriosis^68,69^ and cancer,^9−23^ respectively.

Previous reports show that PFOS induces the proliferation of nontransformed breast and prostate cells but does not induce their transformation into cancer cells, whereas the growth-promoting effects of PFOS on cancer cells and tumors are highly variable.^56−62^ In contrast, CDIMs act as inverse NR4A1 agonists in most solid tumor-derived cell lines and inhibit NR4A1-regulated pro-oncogenic genes/pathways, and in this study, Rh30 and other cancer cells have been used as a “mechanistic model” to investigate whether PFOS is acting as an NR4A1 agonist that enhances NR4A1-mediated pro-oncogenic activities. This approach has its limitations in terms of explaining the complete carcinogenic activity of PFAS since the results show effects on cancer cells and tumors but not on the role of PFOS in the transformation of normal cells.

Results illustrated in Figure 1 demonstrate that PFOS and other polyfluoroalkyl sulfonates directly bind the LBD of NR4A1 using a fluorescent quenching assay as described in previous studies.^65^ Decreased fluorescence was observed for the polyfluoro deca-, nona-, octa-, and heptaalkyl sulfonates (Figure 1), and with the exception of the PFDS congener, these compounds also induced cell proliferation (Figure 4). The reason for this “outlier” effect for PFDS is not known and is being investigated. PFHxS exhibits minimal receptor binding and effects on growth, suggesting that for the linear polyfluoroalkyl sulfonates, the NR4A1-active compounds must have greater than 6 carbons. Previous studies in cancer cell lines showed that low concentrations of PFOS (1–100 nM) induced the proliferation of T47D breast cancer cells; however, the antiestrogen fasoldex inhibited this response, suggesting that the proliferative activity was related to the estrogenic activity of PFOS and PFOS alone did not affect cell growth.^58^ In contrast, 25–200 μM PFOS decreased the growth of A549 lung cancer cells,^57^ and this response would be comparable to that observed for NR4A1 inverse agonists^47,63,64^ (Figure 2). In this study, PFOS induced proliferation of several cancer cell lines by greater than 2-fold, and the fold induction of others varied from <2-fold to minimal induction of cell proliferation effects (Figure 3). The variable responsiveness of these cell lines to the growth-promoting effects of PFOS is not uncommon for other growth-promoting substances; we hypothesized that the variability may be due to PFOS-mediated growth inhibition by activating PPARγ since PFOS binds PPARγ. However, this is unlikely since PPARγ inhibitors do not enhance PFOS-induced cancer cell growth (Figure S1). We are now further investigating the underlying mechanisms causing these cell context-dependent differences observed for inducing cancer cell growth by PFOS. The structure-dependent binding of PFOS and related compounds to NR4A1 and the structure-dependent induction of Rh30 cell proliferation by the hepta-, octa-, and nonafluoroalkyl sulfonates is consistent with a role for NR4A1 in the induction of cell proliferation by these PFAS compounds.

We used the NR4A1-responsive Rh30 cell line as a model to further investigate the role of this receptor in mediating PFOS-induced cell proliferation. Knockdown of NR4A1 by RNA interference resulted in the loss of growth-promoting activity for not only PFOS (Figure 4D) but also the related perfluoroalkyl sulfonates in Rh30 cells after knockdown by NR4A1 (Figure 4E). Thus, the effects of PFOS and related compounds on Rh30 cell proliferation were inversely related to those observed for CDIMs which are inverse NR4A1 agonists that inhibit NR4A1-dependent cell growth.^47,63,64^ The effects of PFOS on other pro-oncogenic and genomic responses that are inhibited by CDIMs were also investigated, and there was an inverse functional relationship between PFOS and CDIMs as NR4A1 ligands. PFOS induced cell migration in a scratch and Boyden chamber assay and induced the NR4A1-responsive PAX3-FOXO1 oncogene and related gene products in Rh30 cells, whereas the opposite effects were previously observed for CDIMs in this cell line.^47,63,64^ Moreover, DIM-3,5-Cl_2_, an NR4A1 inverse agonist, inhibits PFOS-induced growth of Rh30 cells (Figure 6C) and PFOS-induced (NR4A1-dependent) gene products (Figure 6D) in the same cell line. The inverse effects of PFOS vs DIM-3,5-Cl_2_ is also confirmed in a ChIP assay where PFOS had no effect on NR4A1 interactions with the G9a promoter, whereas DIM-3,5-Cl_2_ decreased the interaction of NR4A1 with the transcriptionally active GC-rich region of the G9a promoter.

Rh30 cells were also used as xenografts in athymic nude mice to investigate the effects of PFOS on tumor growth. Higher doses of 20 and 10 mg/kg/day of PFOS inhibited tumor growth, and this was consistent with the results of previous studies on PFOS. However, in mice treated with 0.5 mg/kg/day, there was a significant increase in tumor volume, whereas in mice receiving 0.2 mg/kg/day, tumor growth was not significantly different than the control group, indicating a narrow range of PFOS-enhanced carcinogeneses. Previous reports on the effect of PFOS on tumor growth in rodent models are variable. For example, in genetic mouse models for colon cancer, 10 and 250 mg/kg/day or 200 mg/kg total dose of PFOS decreased intestinal tumor growth,^59,60^ whereas PFOS (10 mg/kg/day) induced tumor growth in athymic nude mice bearing tumorigenic RWPE-2 prostate cancer cells as xenografts.^48^ Differences between studies on the carcinogenicity of PFOS are unknown; however, higher concentrations of PFOS are known to be cytotoxic and this response may be due, in part, to induction of reactive oxygen species (ROS).^57^ In this study using Rh30 cells as a model, there is now evidence that low concentrations and/or doses of PFOS enhance tumorigenesis, and this response is, in part, NR4A1-dependent. The contributions of NR4A1 to other toxicities associated with PFAS are not yet known and are currently being investigated.

## Supporting Information Available

Inhibition of PPARγ does not affect PFOS induction of cancer cell proliferation and this suggests that PPARγ does not influence the growth-promoting effects of PFOS and so probably not associated with cell context-dependent responsiveness to PFOS; growth-promoting effects of PFOS are cell context-dependent; PFOS also binds PPARγ to inhibit cancer cell growth; Is expression of PPARγ in cancer cells the reasons for variable effects of PFOS; Figure S1 showing that PPARγ antagonists (T007, GW9662) do not enhance cancer cell growth-promoting activity of PFOS; and factors other than PPARγ affecting the induction of cancer cell growth by PFOS through NR4A1 (PDF)

## Abbreviations

CDIMs: Bis-indole-derived compounds
DIM-3,5: 1,1-bis(3′-indolyl)-1-(3,5-disubstitutedphenyl)methane
DMSO: dimethyl sulfoxide
LBD: ligand binding domain
NR: nuclear receptor
PFAS: polyfluoroalkyl substances
PFDS: polyfluorodecanesulfonate
pfh_p_s: perfluoroheptanesulfonate
PFH_X_S: perfluorohexanesulfonate
PFNS: perfluorononanesulfonate
PFOA: perfluorooctanoic acid
PFOS: perfluorooctanesulfonate
PPARα: peroxisome proliferator-activated receptor 5
TXND5: thioredoxin domain-containings
XST: linear

## References

[ref1] Wang D. Z.; Goldenman G.; Tugran T.; McNeil A.; Jones M. Per- and polyfluoroalkylether substances: identity, production and use; Nordisk Ministerråd: Copenhagen, 2020.

[ref2] Glüge J.; Scheringer M.; Cousins I. T.; DeWitt J. C.; Goldenman G.; Herzke D.; Lohmann R.; Ng C. A.; Trier X.; Wang Z. An overview of the uses of per- and polyfluoroalkyl substances (PFAS). Environmental science. Processes & impacts 2020, 22 (12), 2345–2373. 10.1039/D0EM00291G. 33125022 PMC7784712

[ref3] Wee S. Y.; Aris A. Z. Environmental impacts, exposure pathways, and health effects of PFOA and PFOS. Ecotoxicology and environmental safety 2023, 267, 115663 10.1016/j.ecoenv.2023.115663. 37976959

[ref4] Evich M. G.; Davis M. J. B.; McCord J. P.; Acrey B.; Awkerman J. A.; Knappe D. R. U.; Lindstrom A. B.; Speth T. F.; Tebes-Stevens C.; Strynar M. J.; Wang Z.; Weber E. J.; Henderson W. M.; Washington J. W. Per- and polyfluoroalkyl substances in the environment. Science 2022, 375 (6580), eabg9065 10.1126/science.abg9065. 35113710 PMC8902460

[ref5] Sun X.; Yang X.; Zhang Y.; Liu Y.; Xiao F.; Guo H.; Liu X. Correlation analysis between per-fluoroalkyl and poly-fluoroalkyl substances exposure and depressive symptoms in adults: NHANES 2005–2018. Science of the total environment 2024, 906, 167639 10.1016/j.scitotenv.2023.167639. 37813256

[ref6] Xu Z.; Du B.; Wang H.; Li Z.; Wu Y.; Wang Q.; Niu Y.; Zhang Q.; Sun K.; Wang J.; Chen S. Perfluoroalkyl substances in umbilical cord blood and blood pressure in offspring: a prospective cohort study. Environm. Health 2023, 22 (1), 72 10.1186/s12940-023-01023-5. PMC10585876 37858165

[ref7] Rosen E. M.; Kotlarz N.; Knappe D. R. U.; Lea C. S.; Collier D. N.; Richardson D. B.; Hoppin J. A. Drinking Water-Associated PFAS and Fluoroethers and Lipid Outcomes in the GenX Exposure Study. Environ. Health Perspect. 2022, 130 (9), 97002 10.1289/EHP11033. 36069575 PMC9450637

[ref8] Radke E. G.; Wright J. M.; Christensen K.; Lin C. J.; Goldstone A. E.; Lemeris C.; Thayer K. A. Epidemiology Evidence for Health Effects of 150 per- and Polyfluoroalkyl Substances: A Systematic Evidence Map. Environ. Health Perspect. 2022, 130 (9), 96003 10.1289/EHP11185. 36178797 PMC9524599

[ref9] Zhang T.; Fu S.; Yu K.; Albanes D.; Moore S. C.; Purdue M. P.; Stolzenberg-Solomon R. Z. Nested Case-Control Studies Investigating Serum Perfluorooctanoate and Perfluorooctane Sulfonate Levels and Pancreatic Ductal Adenocarcinoma in Two Cohorts. Environ. Health Perspect. 2023, 131 (10), 107702 10.1289/EHP13208. 37844029 PMC10578516

[ref10] Li X.; Song F.; Liu X.; Shan A.; Huang Y.; Yang Z.; Li H.; Yang Q.; Yu Y.; Zheng H.; Cao X. C.; Chen D.; Chen K. X.; Chen X.; Tang N. J. Perfluoroalkyl substances (PFASs) as risk factors for breast cancer: a case-control study in Chinese population. Environm. Health 2022, 21 (1), 83 10.1186/s12940-022-00895-3. PMC9463854 36085159

[ref11] Seyyedsalehi M. S.; Boffetta P. Per- and Poly-fluoroalkyl Substances (PFAS) Exposure and Risk of Kidney, Liver, and Testicular Cancers: A Systematic Review and Meta-Analysis. La Medicina del lavoro 2023, 114 (5), e2023040 10.23749/mdl.v114i5.15065. 37878255 PMC10627102

[ref12] van Gerwen M.; Colicino E.; Guan H.; Dolios G.; Nadkarni G. N.; Vermeulen R. C. H.; Wolff M. S.; Arora M.; Genden E. M.; Petrick L. M. Per- and polyfluoroalkyl substances (PFAS) exposure and thyroid cancer risk. EBioMedicine 2023, 97, 104831 10.1016/j.ebiom.2023.104831. 37884429 PMC10667111

[ref13] Madrigal J. M.; Troisi R.; Surcel H. M.; Öhman H.; Kivelä J.; Kiviranta H.; Rantakokko P.; Koponen J.; Medgyesi D. N.; Kitahara C. M.; McGlynn K. A.; Sampson J.; Albert P. S.; Ward M. H.; Jones R. R. Prediagnostic serum concentrations of per- and polyfluoroalkyl substances and risk of papillary thyroid cancer in the Finnish Maternity Cohort. International journal of cancer 2024, 154 (6), 979–991. 10.1002/ijc.34776. 37902275 PMC11286200

[ref14] Chang E. T.; Adami H. O.; Boffetta P.; Cole P.; Starr T. B.; Mandel J. S. A critical review of perfluorooctanoate and perfluorooctanesulfonate exposure and cancer risk in humans. Crit. Rev. Toxicol. 2014, 44 (Suppl 1), 1–81. 10.3109/10408444.2014.905767. 24793953

[ref15] Rodgers K. M.; Udesky J. O.; Rudel R. A.; Brody J. G. Environmental chemicals and breast cancer: An updated review of epidemiological literature informed by biological mechanisms. Environmental research 2018, 160, 152–182. 10.1016/j.envres.2017.08.045. 28987728

[ref16] Stanifer J. W.; Stapleton H. M.; Souma T.; Wittmer A.; Zhao X.; Boulware L. E. Perfluorinated Chemicals as Emerging Environmental Threats to Kidney Health: A Scoping Review. Clinical journal of the American Society of Nephrology: CJASN 2018, 13 (10), 1479–1492. 10.2215/CJN.04670418. 30213782 PMC6218824

[ref17] Bonefeld-Jo̷rgensen E. C.; Long M.; Fredslund S. O.; Bossi R.; Olsen J. Breast cancer risk after exposure to perfluorinated compounds in Danish women: a case-control study nested in the Danish National Birth Cohort. Cancer Causes Control 2014, 25 (11), 1439–1448. 10.1007/s10552-014-0446-7. 25148915 PMC4215104

[ref18] Bonefeld-Jorgensen E. C.; Long M.; Bossi R.; Ayotte P.; Asmund G.; Krüger T.; Ghisari M.; Mulvad G.; Kern P.; Nzulumiki P.; Dewailly E. Perfluorinated compounds are related to breast cancer risk in Greenlandic Inuit: a case control study. Environ. Health 2011, 10, 88 10.1186/1476-069X-10-88. 21978366 PMC3203030

[ref19] Barry V.; Winquist A.; Steenland K. Perfluorooctanoic acid (PFOA) exposures and incident cancers among adults living near a chemical plant. Environ. Health Perspect. 2013, 121 (11–12), 1313–1318. 10.1289/ehp.1306615. 24007715 PMC3855514

[ref20] Shearer J. J.; Callahan C. L.; Calafat A. M.; Huang W. Y.; Jones R. R.; Sabbisetti V. S.; Freedman N. D.; Sampson J. N.; Silverman D. T.; Purdue M. P.; Hofmann J. N. Serum Concentrations of Per- and Polyfluoroalkyl Substances and Risk of Renal Cell Carcinoma. Journal of the National Cancer Institute 2021, 113 (5), 580–587. 10.1093/jnci/djaa143. 32944748 PMC8096365

[ref21] Tsai M. S.; Chang S. H.; Kuo W. H.; Kuo C. H.; Li S. Y.; Wang M. Y.; Chang D. Y.; Lu Y. S.; Huang C. S.; Cheng A. L.; Lin C. H.; Chen P. C. A case-control study of perfluoroalkyl substances and the risk of breast cancer in Taiwanese women. Environ. Int. 2020, 142, 105850 10.1016/j.envint.2020.105850. 32580117

[ref22] Cao L.; Guo Y.; Chen Y.; Hong J.; Wu J.; Hangbiao J. Per-/polyfluoroalkyl substance concentrations in human serum and their associations with liver cancer. Chemosphere 2022, 296, 134083 10.1016/j.chemosphere.2022.134083. 35216980

[ref23] Goodrich J. A.; Walker D.; Lin X.; Wang H.; Lim T.; McConnell R.; Conti D. V.; Chatzi L.; Setiawan V. W. Exposure to perfluoroalkyl substances and risk of hepatocellular carcinoma in a multiethnic cohort. JHEP reports: innovation in hepatology 2022, 4 (10), 100550 10.1016/j.jhepr.2022.100550. 36111068 PMC9468464

[ref24] Su S.; Billy L. J.; Chang S.; Gonzalez F. J.; Patterson A. D.; Peters J. M. The role of mouse and human peroxisome proliferator-activated receptor-α in modulating the hepatic effects of perfluorooctane sulfonate in mice. Toxicology 2022, 465, 153056 10.1016/j.tox.2021.153056. 34861291 PMC10292111

[ref25] Lai T. T.; Eken Y.; Wilson A. K. Binding of Per- and Polyfluoroalkyl Substances to the Human Pregnane X Receptor. Environ. Sci. Technol. 2020, 54 (24), 15986–15995. 10.1021/acs.est.0c04651. 33228354

[ref26] Tachachartvanich P.; Singam E. R. A.; Durkin K. A.; Furlow J. D.; Smith M. T.; La Merrill M. A. In Vitro characterization of the endocrine disrupting effects of per- and poly-fluoroalkyl substances (PFASs) on the human androgen receptor. Journal of hazardous materials 2022, 429, 128243 10.1016/j.jhazmat.2022.128243. 35093747 PMC9705075

[ref27] Behnisch P. A.; Besselink H.; Weber R.; Willand W.; Huang J.; Brouwer A. Developing potency factors for thyroid hormone disruption by PFASs using TTR-TRβ CALUX® bioassay and assessment of PFASs mixtures in technical products. Environ. Int. 2021, 157, 106791 10.1016/j.envint.2021.106791. 34364217

[ref28] De Toni L.; Di Nisio A.; Rocca M. S.; Pedrucci F.; Garolla A.; Dall’Acqua S.; Guidolin D.; Ferlin A.; Foresta C. Comparative Evaluation of the Effects of Legacy and New Generation Perfluoralkyl Substances (PFAS) on Thyroid Cells *In Vitro*. Frontiers in endocrinology 2022, 13, 915096 10.3389/fendo.2022.915096. 35813651 PMC9259843

[ref29] Azhagiya Singam E. R.; Durkin K. A.; La Merrill M. A.; Furlow J. D.; Wang J. C.; Smith M. T. The vitamin D receptor as a potential target for the toxic effects of per- and polyfluoroalkyl substances (PFASs): An in-silico study. Environmental research 2023, 217, 114832 10.1016/j.envres.2022.114832. 36403651 PMC10044465

[ref30] Azhagiya Singam E. R.; Tachachartvanich P.; Fourches D.; Soshilov A.; Hsieh J. C. Y.; La Merrill M. A.; Smith M. T.; Durkin K. A. Structure-based virtual screening of perfluoroalkyl and polyfluoroalkyl substances (PFASs) as endocrine disruptors of androgen receptor activity using molecular docking and machine learning. Environmental research 2020, 190, 109920 10.1016/j.envres.2020.109920. 32795691

[ref31] Qin H.; Niu Y.; Luan H.; Li M.; Zheng L.; Pan Y.; Liu W. Effects of legacy and emerging per- and polyfluoroalkyl substances on PPARα/β/γ regulation and osteogenic/adipogenic differentiation. Environ. Int. 2022, 170, 107584 10.1016/j.envint.2022.107584. 36265359

[ref32] Lai T. T.; Kuntz D.; Wilson A. K. Molecular Screening and Toxicity Estimation of 260,000 Perfluoroalkyl and Polyfluoroalkyl Substances (PFASs) through Machine Learning. J. Chem. Inf. Model. 2022, 62 (19), 4569–4578. 10.1021/acs.jcim.2c00374. 36154169

[ref33] Dale K.; Yadetie F.; Horvli T.; Zhang X.; Frøysa H. G.; Karlsen O. A.; Goksøyr A. Single PFAS and PFAS mixtures affect nuclear receptor- and oxidative stress-related pathways in precision-cut liver slices of Atlantic cod (Gadus morhua). Sci. Total Environ. 2022, 814, 152732 10.1016/j.scitotenv.2021.152732. 34974025

[ref34] Evans N.; Conley J. M.; Cardon M.; Hartig P.; Medlock-Kakaley E.; Gray L. E. Jr In vitro activity of a panel of per- and polyfluoroalkyl substances (PFAS), fatty acids, and pharmaceuticals in peroxisome proliferator-activated receptor (PPAR) alpha, PPAR gamma, and estrogen receptor assays. Toxicology and applied pharmacology 2022, 449, 116136 10.1016/j.taap.2022.116136. 35752307 PMC9341220

[ref35] Almeida N. M. S.; Eken Y.; Wilson A. K. Binding of Per- and Polyfluoro-alkyl Substances to Peroxisome Proliferator-Activated Receptor Gamma. ACS omega 2021, 6 (23), 15103–15114. 10.1021/acsomega.1c01304. 34151090 PMC8210440

[ref36] Kirk A. B.; Michelsen-Correa S.; Rosen C.; Martin C. F.; Blumberg B. PFAS and Potential Adverse Effects on Bone and Adipose Tissue Through Interactions With PPARγ. Endocrinology 2021, 162 (12), bqab194 10.1210/endocr/bqab194. 34480479 PMC9034324

[ref37] Schlezinger J. J.; Puckett H.; Oliver J.; Nielsen G.; Heiger-Bernays W.; Webster T. F. Perfluorooctanoic acid activates multiple nuclear receptor pathways and skews expression of genes regulating cholesterol homeostasis in liver of humanized PPARα mice fed an American diet. Toxicology and applied pharmacology 2020, 405, 115204 10.1016/j.taap.2020.115204. 32822737 PMC7503133

[ref38] Safe S.; Karki K. The Paradoxical Roles of Orphan Nuclear Receptor 4A (NR4A) in Cancer. Molecular cancer research: MCR 2021, 19 (2), 180–191. 10.1158/1541-7786.MCR-20-0707. 33106376 PMC7864866

[ref39] Mohankumar K.; Wright G.; Kumaravel S.; Shrestha R.; Zhang L.; Abdelrahim M.; Chapkin R. S.; Safe S. Bis-indole-derived NR4A1 antagonists inhibit colon tumor and splenic growth and T-cell exhaustion. Cancer immunology, immunotherapy: CII 2023, 72 (12), 3985–3999. 10.1007/s00262-023-03530-3. 37847301 PMC10700478

[ref40] Zhan Y. Y.; Chen Y.; Zhang Q.; Zhuang J. J.; Tian M.; Chen H. Z.; Zhang L. R.; Zhang H. K.; He J. P.; Wang W. J.; Wu R.; Wang Y.; Shi C.; Yang K.; Li A. Z.; Xin Y. Z.; Li T. Y.; Yang J. Y.; Zheng Z. H.; Yu C. D.; Lin S. C.; Chang C.; Huang R. Q.; Lin T.; Wu Q. The orphan nuclear receptor Nur77 regulates LKB1 localization and activates AMPK. Nat. Chem. Biol. 2012, 8 (11), 897–904. 10.1038/nchembio.1069. 22983157

[ref41] Mohankumar K.; Lee J.; Wu C. S.; Sun Y.; Safe S. Bis-Indole-Derived NR4A1 Ligands and Metformin Exhibit NR4A1-Dependent Glucose Metabolism and Uptake in C2C12 Cells. Endocrinology 2018, 159 (5), 1950–1963. 10.1210/en.2017-03049. 29635345 PMC5888234

[ref42] Mohankumar K.; Li X.; Sung N.; Cho Y. J.; Han S. J.; Safe S. Bis-Indole-Derived Nuclear Receptor 4A1 (NR4A1, Nur77) Ligands as Inhibitors of Endometriosis. Endocrinology 2020, 161 (4), bqaa027 10.1210/endocr/bqaa027. 32099996 PMC7105386

[ref43] Zhang L.; Mohankumar K.; Martin G.; Mariyam F.; Park Y.; Han S. J.; Safe S. Flavonoids Quercetin and Kaempferol Are NR4A1 Antagonists and Suppress Endometriosis in Female Mice. Endocrinology 2023, 164 (10), bqad133 10.1210/endocr/bqad133. 37652054 PMC10502789

[ref44] Palumbo-Zerr K.; Zerr P.; Distler A.; Fliehr J.; Mancuso R.; Huang J.; Mielenz D.; Tomcik M.; Fürnrohr B. G.; Scholtysek C.; Dees C.; Beyer C.; Krönke G.; Metzger D.; Distler O.; Schett G.; Distler J. H. Orphan nuclear receptor NR4A1 regulates transforming growth factor-β signaling and fibrosis. Nature medicine 2015, 21 (2), 150–158. 10.1038/nm.3777. 25581517

[ref45] Shrestha R.; Mohankumar K.; Jin U. H.; Martin G.; Safe S. The Histone Methyltransferase Gene G9A Is Regulated by Nuclear Receptor 4A1 in Alveolar Rhabdomyosarcoma Cells. Molecular cancer therapeutics 2021, 20 (3), 612–622. 10.1158/1535-7163.MCT-20-0474. 33277444 PMC7933077

[ref46] Shrestha R.; Mohankumar K.; Martin G.; Hailemariam A.; Lee S. O.; Jin U. H.; Burghardt R.; Safe S. Flavonoids kaempferol and quercetin are nuclear receptor 4A1 (NR4A1, Nur77) ligands and inhibit rhabdomyosarcoma cell and tumor growth. J. Exp. Clin. Cancer Res. 2021, 40 (1), 392 10.1186/s13046-021-02199-9. 34906197 PMC8670039

[ref47] Shrestha R.; Mohankumar K.; Safe S. Bis-indole derived nuclear receptor 4A1 (NR4A1) antagonists inhibit TGFβ-induced invasion of embryonal rhabdomyosarcoma cells. Am. J. Cancer Res. 2020, 10 (8), 2495–2509. 32905449 PMC7471359

[ref48] Hu W. Y.; Lu R.; Hu D. P.; Imir O. B.; Zuo Q.; Moline D.; Afradiasbagharani P.; Liu L.; Lowe S.; Birch L.; Griend D. J. V.; Madak-Erdogan Z.; Prins G. S. Per- and polyfluoroalkyl substances target and alter human prostate stem-progenitor cells. Biochemical pharmacology 2022, 197, 114902 10.1016/j.bcp.2021.114902. 34968493 PMC8890783

[ref49] Imir O. B.; Kaminsky A. Z.; Zuo Q. Y.; Liu Y. J.; Singh R.; Spinella M. J.; Irudayaraj J.; Hu W. Y.; Prins G. S.; Madak Erdogan Z. Per- and Polyfluoroalkyl Substance Exposure Combined with High-Fat Diet Supports Prostate Cancer Progression. Nutrients 2021, 13 (11), 3902 10.3390/nu13113902. 34836157 PMC8623692

[ref50] Yang M.; Su W.; Li H.; Li L.; An Z.; Xiao F.; Liu Y.; Zhang X.; Liu X.; Guo H.; Li A. Association of per- and polyfluoroalkyl substances with hepatic steatosis and metabolic dysfunction-associated fatty liver disease among patients with acute coronary syndrome. Ecotoxicology and environmental safety 2023, 264, 115473 10.1016/j.ecoenv.2023.115473. 37722302

[ref51] Zheng H.; Yin Z.; Luo X.; Zhou Y.; Zhang F.; Guo Z. Association of per- and polyfluoroalkyl substance exposure with metabolic syndrome and its components in adults and adolescents. Environmental science and pollution research international 2023, 30 (52), 112943–112958. 10.1007/s11356-023-30317-x. 37845597 PMC10643431

[ref52] Liu C. Y.; Chen P. C.; Lien P. C.; Liao Y. P. Prenatal Perfluorooctyl Sulfonate Exposure and Alu DNA Hypomethylation in Cord Blood. International journal of environmental research and public health 2018, 15 (6), 1066 10.3390/ijerph15061066. 29795014 PMC6025582

[ref53] Pierozan P.; Cattani D.; Karlsson O. Perfluorooctane sulfonate (PFOS) and perfluorooctanoic acid (PFOA) induce epigenetic alterations and promote human breast cell carcinogenesis in vitro. Archives of toxicology 2020, 94 (11), 3893–3906. 10.1007/s00204-020-02848-6. 32700164 PMC7603464

[ref54] Petroff R. L.; Cavalcante R. G.; Langen E. S.; Dolinoy D. C.; Padmanabhan V.; Goodrich J. M. Mediation effects of DNA methylation and hydroxymethylation on birth outcomes after prenatal per- and polyfluoroalkyl substances (PFAS) exposure in the Michigan mother-infant Pairs cohort. Clin. Epigenetics 2023, 15 (1), 49 10.1186/s13148-023-01461-5. 36964604 PMC10037903

[ref55] Guerrero-Preston R.; Goldman L. R.; Brebi-Mieville P.; Ili-Gangas C.; Lebron C.; Witter F. R.; Apelberg B. J.; Hernández-Roystacher M.; Jaffe A.; Halden R. U.; Sidransky D. Global DNA hypomethylation is associated with in utero exposure to cotinine and perfluorinated alkyl compounds. Epigenetics 2010, 5 (6), 539–546. 10.4161/epi.5.6.12378. 20523118 PMC3322495

[ref56] Pierozan P.; Karlsson O. PFOS induces proliferation, cell-cycle progression, and malignant phenotype in human breast epithelial cells. Archives of toxicology 2018, 92 (2), 705–716. 10.1007/s00204-017-2077-8. 29063134 PMC5818598

[ref57] Mao Z.; Xia W.; Wang J.; Chen T.; Zeng Q.; Xu B.; Li W.; Chen X.; Xu S. Perfluorooctane sulfonate induces apoptosis in lung cancer A549 cells through reactive oxygen species-mediated mitochondrion-dependent pathway. Journal of applied toxicology: JAT 2013, 33 (11), 1268–1276. 10.1002/jat.2785. 22976841

[ref58] Sonthithai P.; Suriyo T.; Thiantanawat A.; Watcharasit P.; Ruchirawat M.; Satayavivad J. Perfluorinated chemicals, PFOS and PFOA, enhance the estrogenic effects of 17β-estradiol in T47D human breast cancer cells. Journal of applied toxicology: JAT 2016, 36 (6), 790–801. 10.1002/jat.3210. 26234195

[ref59] Wimsatt J.; Villers M.; Thomas L.; Kamarec S.; Montgomery C.; Yeung L. W.; Hu Y.; Innes K. Oral perfluorooctane sulfonate (PFOS) lessens tumor development in the APC^min^ mouse model of spontaneous familial adenomatous polyposis. BMC Cancer 2016, 16 (1), 942 10.1186/s12885-016-2861-5. 27927180 PMC5143440

[ref60] Wimsatt J. H.; Montgomery C.; Thomas L. S.; Savard C.; Tallman R.; Innes K.; Jrebi N. Assessment of a mouse xenograft model of primary colorectal cancer with special reference to perfluorooctane sulfonate. PeerJ. 2018, 6, e5602 10.7717/peerj.5602. 30405966 PMC6216948

[ref61] Pesonen M.; Vähäkangas K. Involvement of per- and polyfluoroalkyl compounds in tumor development. Archives of toxicology 2024, 98 (5), 1241–1252. 10.1007/s00204-024-03685-7. 38478087 PMC10965717

[ref62] Solan M. E.; Lavado R. The use of in vitro methods in assessing human health risks associated with short-chain perfluoroalkyl and polyfluoroalkyl substances (PFAS). Journal of applied toxicology: JAT 2022, 42 (8), 1298–1309. 10.1002/jat.4270. 34873727

[ref63] Lacey A.; Hedrick E.; Li X.; Patel K.; Doddapaneni R.; Singh M.; Safe S. Nuclear receptor 4A1 (NR4A1) as a drug target for treating rhabdomyosarcoma (RMS). Oncotarget 2016, 7 (21), 31257–31269. 10.18632/oncotarget.9112. 27144436 PMC5058754

[ref64] Lacey A.; Rodrigues-Hoffman A.; Safe S. PAX3-FOXO1A Expression in Rhabdomyosarcoma Is Driven by the Targetable Nuclear Receptor NR4A1. Cancer research 2017, 77 (3), 732–741. 10.1158/0008-5472.CAN-16-1546. 27864345 PMC5290192

[ref65] Lee S. O.; Li X.; Hedrick E.; Jin U. H.; Tjalkens R. B.; Backos D. S.; Li L.; Zhang Y.; Wu Q.; Safe S. Diindolylmethane analogs bind NR4A1 and are NR4A1 antagonists in colon cancer cells. Molecular endocrinology (Baltimore, Md.) 2014, 28 (10), 1729–1739. 10.1210/me.2014-1102. 25099012 PMC4179635

[ref66] Jabeen M.; Fayyaz M.; Irudayaraj J. Epigenetic Modifications, and Alterations in Cell Cycle and Apoptosis Pathway in A549 Lung Carcinoma Cell Line upon Exposure to Perfluoroalkyl Substances. Toxics 2020, 8 (4), 112 10.3390/toxics8040112. 33238432 PMC7711517

[ref67] Lee S. O.; Andey T.; Jin U. H.; Kim K.; Singh M.; Safe S. The nuclear receptor TR3 regulates mTORC1 signaling in lung cancer cells expressing wild-type p53. Oncogene 2012, 31 (27), 3265–3276. 10.1038/onc.2011.504. 22081070 PMC3299891

[ref68] Ao J.; Zhang R.; Huo X.; Zhu W.; Zhang J. Environmental exposure to legacy and emerging per- and polyfluoroalkyl substances and endometriosis in women of childbearing age. Science of the total environment 2024, 907, 167838 10.1016/j.scitotenv.2023.167838. 37839491

[ref69] Hammarstrand S.; Jakobsson K.; Andersson E.; Xu Y.; Li Y.; Olovsson M.; Andersson E. M. Perfluoroalkyl substances (PFAS) in drinking water and risk for polycystic ovarian syndrome, uterine leiomyoma, and endometriosis: A Swedish cohort study. Environ. Int. 2021, 157, 106819 10.1016/j.envint.2021.106819. 34391986

