# Supplementary material for: Perfluorooctane Sulfonate (PFOS) and Related Compounds Induce Nuclear Receptor 4A1 (NR4A1)-Dependent Carcinogenesis
Source: Chem Res Toxicol. 2025 Mar 11;38(4):705–16. doi: 10.1021/acs.chemrestox.4c00528 (PMC12015964; doi:10.1021/acs.chemrestox.4c00528)
Supplement: Supplementary file 1 — tx4c00528_si_001.pdf [file tx4c00528_si_001.pdf]

SUPPORTING INFORMATION:

PERFLUOROOCTANE SULFONATE (PFOS) AND RELATED COMPOUNDS INDUCE NUCLEAR RECEPTOR 4A1 (NR4A1)-DEPENDENT CARCINOGENESIS

Amanuel Hailemariam[1] , Srijana Upadhyay[1] , Vinod Srivastava[2] , Zahin Hafiz [1] , Lei Zhang[1] , Wai Ning Tsui[1] , Arafat Rahman Oany[1] , Jaileen Rivera-Rodriguez [3] , Robert S. Chapkin[3] , Nicole Riddell [4] , Robert McCrindle[4] , Alan McAlees [4] and Stephen Safe[1]*

[1] Department of Veterinary Physiology and Pharmacology, College of Veterinary Medicine, Texas A&M University, College Station, TX 77843 USA

[2] Department of Veterinary Integrative Biosciences, Texas A&M University, College Station, TX 77845 USA

[3] Department of Nutrition, Program in Integrative Nutrition and Complex Diseases, Texas A&M University, College Station, TX 77843 USA

[4] Wellington Laboratories Inc, 345 Southgate Dr., Guelph, ON N1G 3M5 Canada

*Corresponding Author: Stephen Safe, Email: ssafe@cvm.tamu.edu; Tel. (979) 845-5988; Fax. (979) 862-4929.

Table of Contents

Cover Page ……………………………………………………………………………………...pg 1

Table of Contents …………………………………………………………………………………pg 1

Supplemental Figure 1 ……………………………………………………………………………pg 2

Figure S1

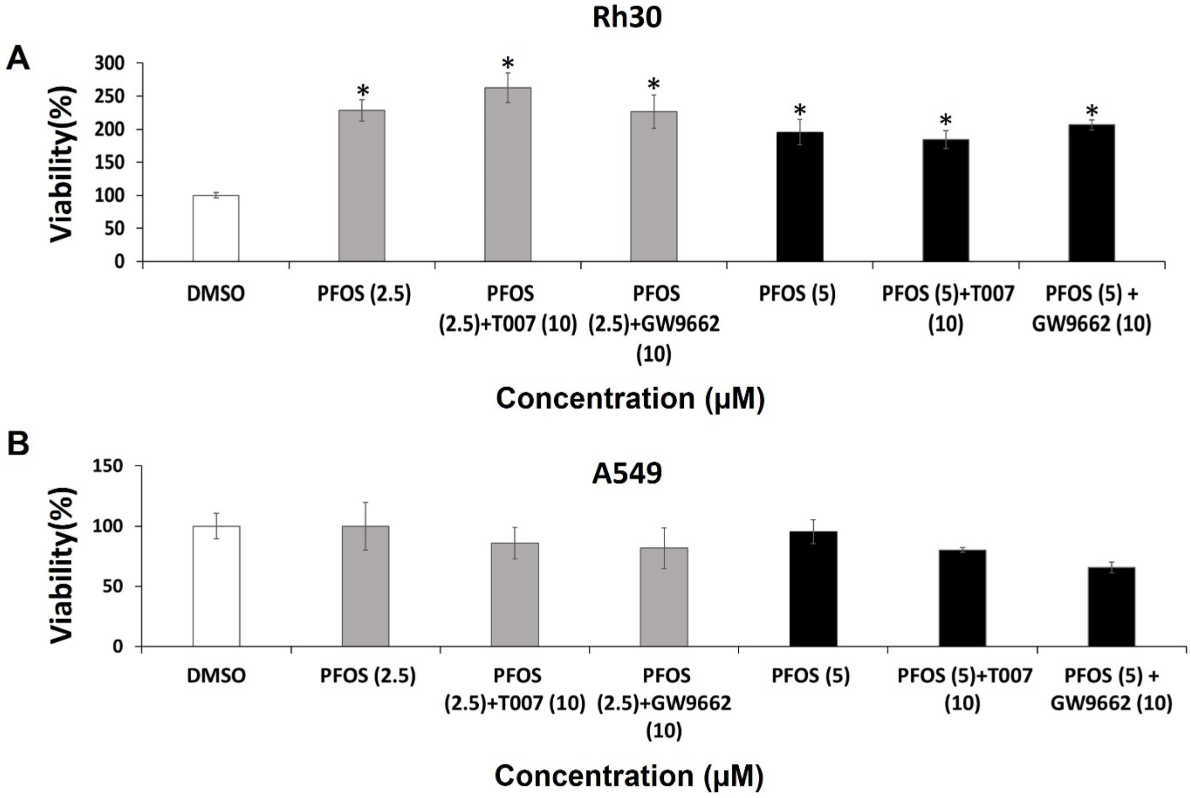
Rh30
A
300
250
200
150
100
50
0
Viability(%)
DMSO
PFOS (2.5)
PFOS (2.5)+T007 (10)
PFOS (2.5)+GW9662 (10)
PFOS (5)
PFOS (5)+T007 (10)
PFOS (5) + GW9662 (10)
Concentration (μM)
B
A549
150
100
50
0
Viability(%)
DMSO
PFOS (2.5)
PFOS (2.5)+T007 (10)
PFOS (2.5)+GW9662 (10)
PFOS (5)
PFOS (5)+T007 (10)
PFOS (5) + GW9662 (10)
Concentration (μM)
